# Wahrnehmungen zum Umgang mit Opioiden bei COVID-19: Eine Umfrage unter Mitgliedern der Deutschen Gesellschaft für Palliativmedizin

**DOI:** 10.1007/s00482-021-00596-9

**Published:** 2021-10-18

**Authors:** Vera Peuckmann-Post, Alexandra Scherg, Norbert Krumm, Carolin Hagedorn, Lukas Radbruch, Andras Keszei, Roman Rolke, Frank Elsner

**Affiliations:** 1 Klinik für Palliativmedizin, Medizinische Fakultät, RWTH Aachen University, Pauwelsstr 30, 52074 Aachen, Deutschland; 2 Abteilung für Hämatologie/Onkologie, Evangelisches Krankenhaus Wesel, Wesel, Deutschland; 3 Klinik und Poliklinik für Frauenheilkunde, Universitätsklinikum Leipzig, Leipzig, Deutschland; 4 Klinik für Palliativmedizin, Universitätsklinikum Bonn, Bonn, Deutschland; 5 Center for Translational & Clinical Research, Medizinische Fakultät, RWTH Aachen University, Aachen, Deutschland

**Keywords:** Opioide, Morphin, COVID-19, Symptomkontrolle, Palliativmedizin, Opioid, Morphine, COVID-19, Symptom control, Palliative care

## Abstract

**Hintergrund:**

Obwohl Opioide wirksam Schmerzen und Dyspnoe lindern, findet dies in Leitlinien zur Symptomkontrolle unterschiedliche Gewichtung. Dies kann zu Unsicherheiten bezüglich Indikationen und ethischer Implikationen im Umgang mit Opioiden auch bei COVID-19 führen.

**Ziel der Arbeit:**

Wir untersuchten bei Mitgliedern der Deutschen Gesellschaft für Palliativmedizin (DGP) die persönliche Wahrnehmung des Umgangs mit Morphin/Opioiden (M/O) zur Symptomkontrolle *innerhalb *und *außerhalb* der Palliativmedizin (PM), auch bei der Betreuung COVID-19-Erkrankter.

**Material und Methoden:**

Mittels Survey Monkey® wurden DGP-Mitglieder anonymisiert nach ihrer eigenen Wahrnehmung des Umgangs mit M/O zur Symptomkontrolle befragt.

**Ergebnisse und Diskussion:**

Von den 6192 DGP-Mitgliedern nahmen N = 506 teil. Den Umgang mit M/O *innerhalb *der PM beschrieben 98 % der befragten Ärzt:innen und Pflegekräfte als „sicher und vertraut“ bzw. 95 % als „klar geregelt“, während dies für die Bereiche *außerhalb *der PM von weniger als der Hälfte angegeben wurde (48 %/38 %). Bei der Betreuung COVID-19-Erkrankter wurde der Umgang mit M/O *außerhalb *der PM noch seltener als „sicher und vertraut“ (26 %) oder „klar geregelt“ (23 %) wahrgenommen. Dyspnoe (99 %/52 %), Erleichterung des Sterbeprozesses (62 %/37 %), Unruhe (30 %/15 %) und Angst/Panik (27 %/13 %) wurden häufiger *innerhalb *als* außerhalb *der PM als allgemeine Indikationen genannt. 89 % der Befragten wünschten sich die Einbindung eines PM-Konsilteams.

**Schlussfolgerung:**

Mitglieder der DGP nahmen deutliche Unsicherheiten im Umgang mit M/O *außerhalb *der PM wahr. Einheitliche interdisziplinäre Leitlinien zur Symptomkontrolle etwa bei Dyspnoe, mehr Lehre und die Einbindung eines PM-Konsilteams sollten zukünftig mehr bedacht werden.

## Abstract (en)

**Background:**

Opioids efficiently alleviate pain and dyspnea. However, guidelines on symptom management with opioids differ, which may lead to an uncertainty concerning opioid indication and ethical implication among medical staff, especially when caring for COVID-19 patients.

**Aims:**

We aimed to examine the perception of members of the German Association for Palliative Medicine (DGP) concerning the administration of morphine as the gold standard opioid (subsequently termed M/O) for symptom control *within *and *outside *of a palliative care (PC) setting, including care for COVID-19 patients.

**Methods:**

DGP members received an anonymized online questionnaire (Survey Monkey®) containing questions regarding their perception of symptom management with M/O in general and in particular concerning COVID-19 patients. Participants were asked to rate their perception *within *and *outside* of a PC setting.

**Results:**

Of the 6129 DGP members, N = 506 participated. DGP physicians and nurses perceived handling of M/O as “certain and confident” (98%) and “clearly regulated” *within PC* (95%) but rated it significantly lower for *outside* *PC* (48%/38%). When caring for COVID-19 patients, handling of M/O was even less often rated “certain and confident” (26%) or “clearly regulated” (23%) for *outside *PC. Dyspnea (99%/52%), relief from the dying process (62%/37%), restlessness (30%/15%) and fear or panic (27%/13%) were more frequently rated as general indications for morphine *within *versus* outside *PC. Most participants (89%) wished to involve palliative care consultation teams.

**Conclusions:**

DGP members perceived substantial uncertainty in the handling of M/O for medical fields *outside *PC. Uniform interdisciplinary guidelines for symptom control, more education, and involvement of a PC consultation team should be increasingly considered in the future.

## Hintergrund und Fragestellung

Opioide lindern wirksam Schmerzen und Dyspnoe. Leitlinien zu Opioiden bei Dyspnoe divergieren jedoch stark innerhalb von Disziplinen wie Palliativmedizin (PM), Pneumologie und Intensivmedizin [21, 22, 26, 30]. In der „S3 Leitlinie – Empfehlungen zur stationären Therapie von Patienten mit COVID-19“ wurden bis zur dritten Pandemiewelle „Symptomkontrolle“, „Morphin“ oder „Opioide“ nicht erwähnt [20]. Teils polarisierende Auffassungen in den Medien über Intensivmedizin und Sterberisiko [11] sowie die Anklage eines Oberarztes wegen Totschlags an COVID-19 erkrankten Patienten [5] veranschaulichen eine teils brisante Situation.

Wir untersuchten daher Wahrnehmungen zum Umgang mit Morphin/Opioiden (M/O) zur Symptomkontrolle *innerhalb* und *außerhalb* der PM, allgemein und bei COVID-19.

## Studiendesign und Untersuchungsmethoden

Auf Grundlage von Interviews mit Ärzten und Pflegekräften innerhalb wie außerhalb der Palliativmedizin (Einzelinterviews und Fokusgruppeninterview, nicht publiziert) sowie auf der Grundlage internationaler Literatur identifizierten wir häufige Fragestellungen und Assoziationen zum Umgang mit Opioiden in der Symptomkontrolle. Mitgliedern der Deutschen Gesellschaft für Palliativmedizin (DGP) wurde per E‑Mail ein Link zu der anonymisierten Online-Umfrage (Survey Monkey®) zugesandt mit der Bitte um Beantwortung dieser vom 14.09.–11.10.2020. Der Fragebogen beinhaltete soziodemografische Daten und Fragen zur Wahrnehmung der klinischen Anwendung von Opioiden allgemein und speziell bei COVID-19 Erkrankten.

Wir erläuterten im Fragebogen: „Basierend auf klinischen Erfahrungsberichten sowie internationaler Literatur entsteht der Eindruck, dass der Umgang mit Opioiden wie Morphin zur Symptomkontrolle mit Unsicherheit behaftet sein kann. Daher möchten wir eine Umfrage unter den Mitgliedern der DGP zum Umgang mit Opioiden durchführen. Wir sind an Ihren eigenen Erfahrungen interessiert, aber auch daran, wie Sie Kolleginnen und Kollegen anderer Fachrichtungen im Umgang mit Opioiden zur Symptomlinderung wahrnehmen. […] „Morphin“ soll dabei exemplarisch für die Gruppe der Opioide genannt werden.“

Wir erklärten, dass wir in dieser Untersuchung die Fragen „zwischen dem palliativmedizinischen Bereich (z. B. Palliativstation oder ambulanter Palliativdienst) und der Anwendung in anderen Fachkliniken (z. B. Innere Medizin, Chirurgie, Orthopädie/Unfallchirurgie, Neurologie, Intensivstationen etc.)“ unterscheiden. Diese Unterscheidung bezeichnen wir hier nachfolgend als „*innerhalb* der PM“ und „*außerhalb* der PM“.

Zur Einschätzung setzten wir auch eine sechsstufige Likert-Skala ein („stimme gar nicht zu“ bis „stimme voll zu“). In der Aufbereitung der Daten wurden die ersten drei Grade rot („stimme nicht zu“) und die letzten drei Grade blau („stimme zu“) zur Veranschaulichung hinterlegt.

Um die Antworten der Umfrageteilnehmer in Bezug auf *innerhalb* und *außerhalb* der Palliativmedizin zu vergleichen, analysierten wir marginale mittlere Antwortwerte für paarweise Beobachtungen unter Verwendung der Scores 1 bis 6 [2]. Wir präsentieren Schätzungen der Unterschiede der marginalen Mittelwerte und entsprechende 95 %-Wald-Konfidenzintervalle. Die zuständige Ethikkommission des Uniklinikums Aachen genehmigte die Durchführung der Studie (EK 303/20).

## Ergebnisse

### Drop-out-Analyse

Von den 6192 DGP-Mitgliedern^1^ nahmen 506 an der Online-Umfrage teil (Antwortrate 8,2 %). Nach ihrer überwiegenden beruflichen Tätigkeit gefragt, gaben 282 Teilnehmer (TN) an, Ärzt:innen zu sein, 164 waren Pflegekräfte, 21 gehörten anderen Berufsgruppen an (4 Psycholog:innen, 6 Sozialarbeiter:innen, 5 Physiotherapeut:innen und 6 Seelsorgende). 37 TN machten keine Angabe.

Ein Vergleich der soziodemografischen Struktur ergab eine vergleichbare Aufteilung von Umfrage-TN zu allen DGP-Mitgliedern: Männer 33 %/37 %, Frauen 66 %/62 %; Alter 18–30 Jahre: 3 %/1 %, 31–40 Jahre: 10 %/9 %, 41–50 Jahre: 29 %/23 %, 51–60 Jahre: 42 %/38 %.

Der Anteil der Berufe bei den Umfrage TN/DGP Mitgliedern war ebenfalls sehr ähnlich: Ärzt:innen 55 %/55 %; Pflege 32 %/29 %; Soziale Arbeit 1 %/3 %; Psychologie 1 %/2 %; Seelsorge 1 %/2 %; Physiotherapie 1 %/Physio-Ergo-Logotherapie (letztere beiden in DGP-Datei gemeinsam erfasst) 2 %.

### Wahrnehmung des Umgangs mit M/O

Für Fragen zur klinischen Anwendung von M/O werden in diesem Artikel primär die Daten von Ärzt:innen und Pflegekräften gezeigt, da diese Berufsgruppen Opioide primär anwenden.

**Figure 1:**
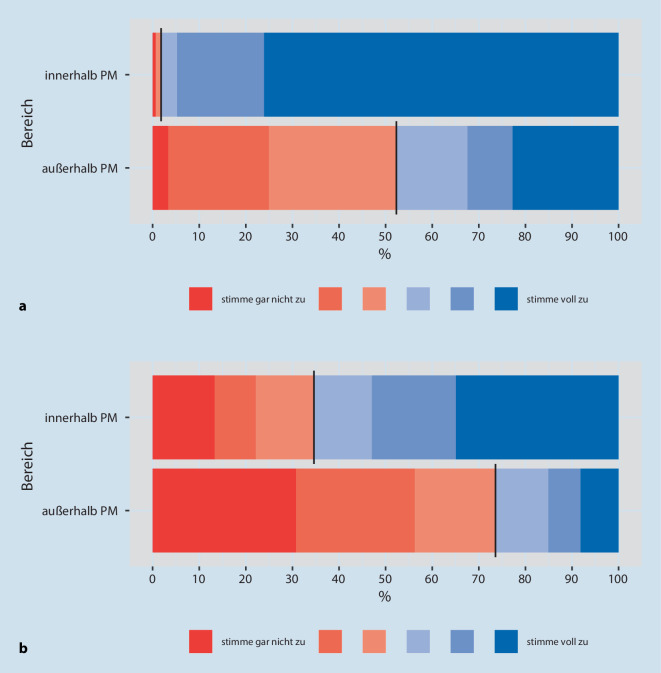

Unter den Ärzt:innen und Pflegekräften schätzten den Umgang mit M/O nahezu alle (98 %) für den *PM Bereich* als sicher und vertraut ein, jedoch nur zur Hälfte (48 %) für den Bereich *außerhalb *der PM (Abb. 1a, Grade 4–6/6). Je älter die Ärzt:innen und Pflegekräfte waren, umso häufiger stimmten sie der Frage nach sicherem Umgang im *PM-Bereich* „voll zu“ (Grad 6/6) –30 Jahre 60 %, 31–40 Jahre 67 %, 41–50 Jahre: 73 %, 51–60 Jahre:78 %, > 60 Jahre 87 % (nicht in der Abb.).

Betrachtet man die Angaben sämtlicher Umfrage-TN (inklusive Physiotherapeut:innen, Psycholog:innen, Sozialarbeiter:innen), so stimmten diese ebenfalls zu 99 % einem sicheren Umgang *innerhalb *der PM zu, jedoch für den Bereich *außerhalb *der PM noch 4 % weniger (gesamte TN = 44 %) als bei der Auswertung der Angaben von Ärzt:innen und Pflegekräften. Dabei übersprangen acht TN die Frage (nicht in der Abb.).

### Wahrnehmungen zur Klarheit der Regelung einer M/O-Gabe

**Figure 2:**
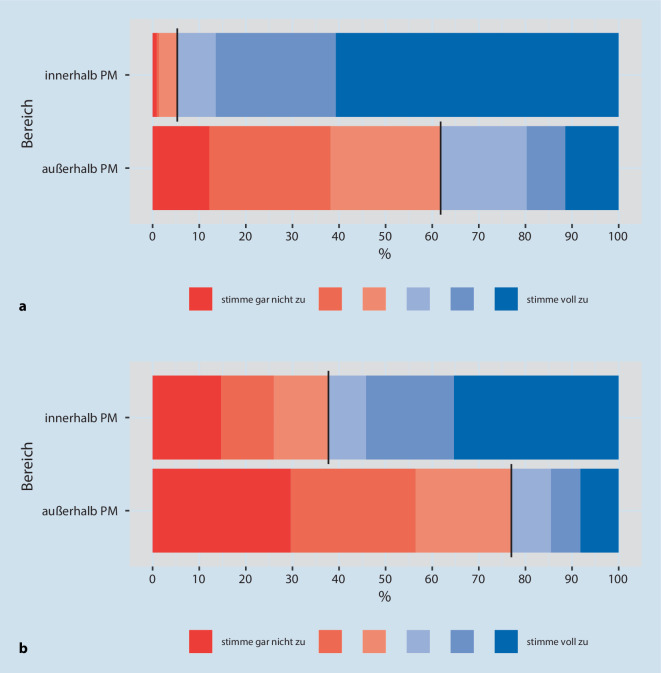

Eine klare Regelung für die Gabe von M/O sahen ebenfalls fast alle (95 %) für den *PM-Bereich*, aber nur 38 % für den Bereich *außerhalb *der PM (Abb. 2a, Grade 4–6/6).

*Bezogen auf COVID-19 Erkrankte *beurteilten etwa ein Drittel weniger Ärzt:innen und Pflegekräfte den Umgang mit M/O *innerhalb *der PM als sicher (65 %) bzw. die Regelung als klar (62 %), aber für den Bereich *außerhalb *der PM nur jeder vierte als sicher (26 %) bzw. klar geregelt (23 %) (Abb. 1b und 2b, jeweils Grade 4–6/6).

**Figure 3:**
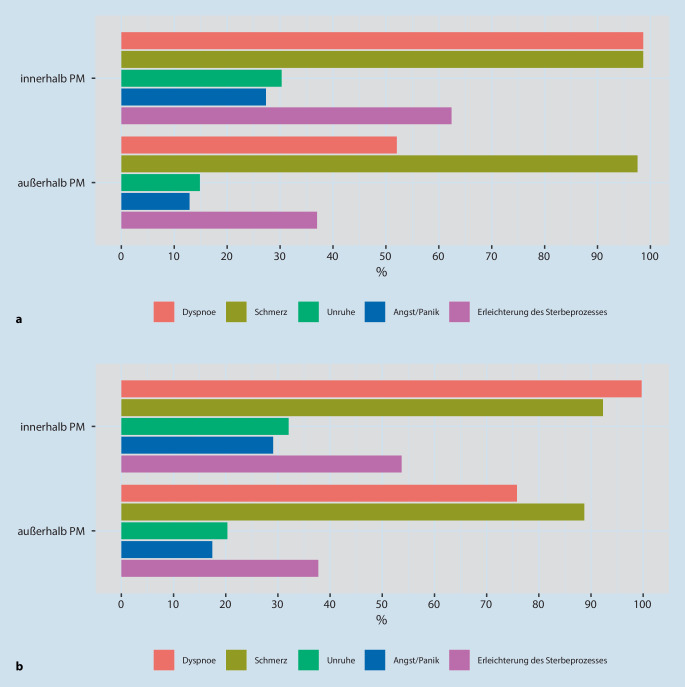

Die häufigsten allgemeinen Indikationen für die Gabe von M/O waren „Schmerzen“ und „Dyspnoe“ (je 99 %) *innerhalb *der PM (Abb. 3a), gefolgt von „Erleichterung des Sterbeprozesses“ (62 %), „Unruhe“ (30 %) und „Angst/Panik“ (27 %). Erneut schätzte nur etwa die Hälfte der Ärzt:innen und Pflegekräfte ihre Kolleg:innen *außerhalb* der PM so ein, dass für diese die Symptome „Dyspnoe“ (52 %), „Erleichterung des Sterbeprozesses“ (37 %), „Unruhe“ (15 %) und „Angst/Panik“ (13 %) als M/O Indikation gelten würden.

Die Indikationen Dyspnoe, Unruhe, Angst/Panik wurden im Vergleich zum Bereich *innerhalb *der PM zwar insgesamt seltener *außerhalb *der PM wahrgenommen, jedoch *außerhalb *der PM *bei COVID-19 Erkrankten* häufiger als bei den allgemeinen Angaben (Abb. 3a, b).

**Figure 4:**
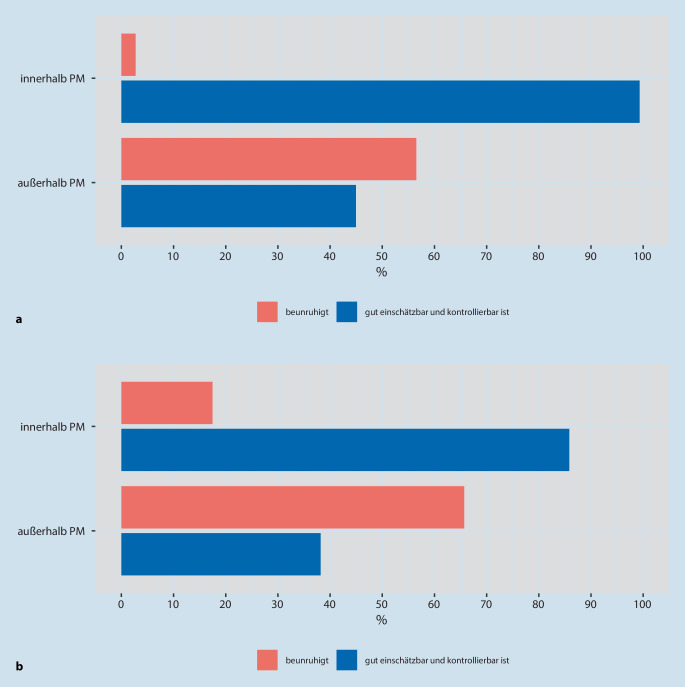

Ebenso beurteilten nahezu alle Ärzt:innen und Pflegekräfte (99 %) eine Atemdepression als unerwünschte Wirkung von M/O *innerhalb *der PM als gut einschätzbar und kontrollierbar. Jedoch hielten nur etwa die Hälfte (45 %) der Opioidanwendenden diese Atemdepression *außerhalb *der PM für gut einschätzbar und kontrollierbar (Abb. 4a).

Bezogen auf *COVID-19 Erkrankte,* wurde jeweils *innerhalb und außerhalb *der PM etwas häufiger geschätzt, dass Atemdepression eine unerwünschte Wirkung von M/O ist, die beunruhigt (17 %/66 %) (Abb. 4b).

### Wahrnehmung zum Einsatz von M/O beim Sterben

**Figure 5:**
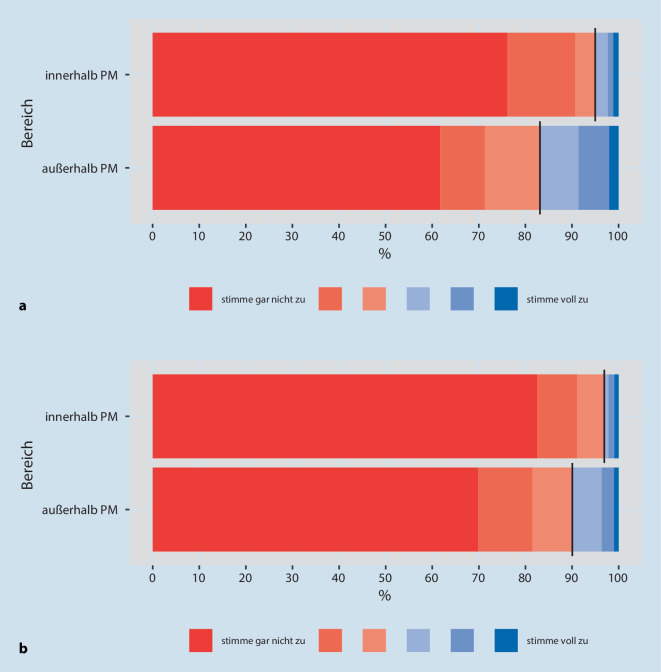

Die TN Mehrheit negierte, dass M/O *innerhalb und außerhalb *der PM gezielt eingesetzt werde(n), um das Sterben zu beschleunigen (95 %/83 %). Die meisten der TN, die jedoch zustimmten, gaben dies mit 17 % für den Bereich *außerhalb *der PM an (ohne COVID-19-Bezug). A*ußerhalb* der PM empfanden nur 10 % der TN den M/O-Einsatz bei der Behandlung* COVID-19 Erkrankter* als Beschleunigung des Sterbens (Abb. 5a, b, Grade 4–6/6).

Diejenigen TN, die für die Bereiche *innerhalb* (5 %) wie *außerhalb* (17 %) der PM angaben, M/O werde(n) gezielt eingesetzt, um das Sterben zu beschleunigen, waren überwiegend Ärzt:innen, die über 20 Jahre Erfahrung hatten, im Krankenhaus tätig sowie älter als 50 Jahre waren.

### Wahrnehmung zu bevorzugten Applikationswegen bei der Anwendung von M/O

Als bevorzugte Applikationsformen von M/O wurde überwiegend die orale Gabe angegeben (*innerhalb/außerhalb PM:* 80 %/76 %), gefolgt von intravenös (iv, 51 %/37 %), subkutan (sc, 88 %/36 %) und transdermal (47 %/49 %).

Gewünscht wurde von allen TN allgemein vor allem die „(bessere) Einbindung eines palliativmedizinischen Konsilteams“ (89 %), gefolgt von „(mehr) Lehre (77 %)“, „(mehr) Teamkonferenzen“ (76 %) und „(mehr) Supervisionen“ (71 %). Hier wurde nicht zwischen den Bereichen *innerhalb* und *außerhalb der PM* unterschieden.

### Vergleich der Bereiche *innerhalb* versus *außerhalb* der PM

Vergleicht man die Wahrnehmungen für die Bereiche *innerhalb* versus *außerhalb* der PM, so stimmten die TN eher für den Bereich *innerhalb* der PM zu im Hinblick auf folgende Fragen: „Die Indikation für die Gabe von Morphin ist klar und einheitlich geregelt“ (Unterschied des Durchschnittswerts [innerhalb der PM – außerhalb der PM] 2,2; 95 % Konfidenzintervall 2,1; 2,4) und „Der Umgang mit Morphin ist sicher und vertraut“ (1,9; 1,8; 2,1).

Diese Unterschiede fielen etwas schwächer bei den Fragen mit *COVID-19* Bezug aus, aber weiterhin stimmten TN eher für den Bereich *innerhalb* der PM zu, verglichen mit *außerhalb* der PM: „Die Indikation für die Gabe von Morphin bei *COVID-19 Erkrankten* ist klar und einheitlich geregelt“ (1,5; 1,3; 1,7), und „Der Umgang mit Morphin bei *COVID-19 Erkrankten* ist sicher und vertraut“ (1,5; 1,4; 1,7).

Vergleicht man die Fragen „Morphin wird gezielt eingesetzt um das Sterben zu beschleunigen“ (−0,6; −0,7; −0,4) und „Morphin wird bei *COVID-19 Erkrankten* gezielt eingesetzt um das Sterben zu beschleunigen“ (−0,3; −0,4; −0,2), so stimmten die TN eher für den Bereich *außerhalb* der PM zu.

## Diskussion

### Heterogener Stellenwert von Opioiden in Leitlinien

Opioide sind für den Einsatz bei starken Schmerzen zugelassen [6], während die Anwendung bei Dyspnoe off-label ist. Die palliativmedizinische S3-Leitlinie für Menschen mit nicht mehr heilbarer Krebserkrankung empfiehlt Opioide klar zur Therapie von u. a. Schmerz und Dyspnoe (Atemnot) [22].

Während Rosenbruch et al. in ihrer „SOP – Atemnot bei erwachsenen Patienten“ bei fehlendem Ansprechen auf allgemeine, nichtpharmakologische Maßnahmen ebenfalls die Gabe von Opioiden und ggf. Benzodiazepinen empfehlen [28], sind andere Leitlinien zur Symptomkontrolle von Dyspnoe mit Opioiden zurückhaltend formuliert [30] oder nennen Opioide hierzu gar nicht [20, 21]: In der „S3-Leitlinie – Empfehlungen zur stationären Therapie von Patienten mit COVID-19“ wurde Palliativmedizin bis zum 16.05.2021 vor allem im Sinne von Therapiebegrenzung genannt [20]. Zwar wurde festgestellt: „Der Palliativversorgung mit dem Ziel der optimalen Linderung von belastenden Symptomen wie Dyspnoe, Husten, Schwäche und Fieber, Angst, Panik, Unruhe und Delir kommt in diesen Situationen eine besondere Bedeutung zu“, jedoch fehlten konkrete Empfehlungen zur Symptomkontrolle mit Opioiden – wie sie in separaten Handlungsempfehlungen der Deutschen Gesellschaft für Palliativmedizin dargestellt waren [26]. Dosierungsbeispiele zur Symptomkontrolle mit Opioiden fanden in die S3-Leitlinie erst während der dritten COVID-19 Welle am 17.05.2021 Eingang, dann unter Beteiligung der Deutschen Gesellschaft für Palliativmedizin [19].

Die im Jahr 2005 publizierten „Empfehlungen zur Behandlung respiratorischer Komplikationen bei einer Viruspandemie“ der Deutschen Gesellschaft für Pneumologie nannten Opioide nicht zur Symptomkontrolle der Dyspnoe, sondern nur bezüglich „stärkerer Schmerzen“ und der „Einleitung der Beatmung“ [21]. Die Empfehlungen der aktuelleren COPD-Leitlinie warnen vor „bedeutsame(n), unerwünschte(n) Effekten, insbesondere der Atemdepression“, weswegen „der Einsatz auf wenige besonders beeinträchtigte Patienten mit schwerer Atemnot beschränkt“ sein solle [30]. Ein aktuelles systematisches Review zur Behandlung von Atemnot bei Patient:innen mit fortgeschrittener Krebserkrankung fand keine Assoziation zwischen pharmakologischen Substanzen und erhöhter Wirksamkeit gegenüber Placebo [15]. Die Autoren bewerteten viel der Evidenz mit „niedriger Qualität“ und betonten, dass zukünftige Studien Atemnot als multidimensionales Geschehen messen sollten.

### Stellenwert von Opioiden bei Dyspnoe und COVID-19

Die Diagnose und Therapie von Luftnot und Ateminsuffizienz sind komplex [8]. Die medizinische Versorgung überwiegend älterer Patient:innen mit teils lebenslimitierenden Komorbiditäten beinhaltet internistische und intensivmedizinische Interventionen bis hin zu Intubation und Beatmung. Dabei nimmt die angemessene Symptombehandlung, insbesondere von Luftnot, eine Schlüsselrolle ein [3, 7, 14, 17, 23, 26]. Palliativmedizinisch betreute Patienten mit COVID-19 zeigten in der aktuellen Literatur insbesondere Dyspnoe und Agitation als führende Symptome, die meist erfolgreich mit relativ niedrig dosierten Opioiden und Benzodiazepinen kontrolliert werden konnten [3, 17, 23]. Der Grad der Dyspnoe schien unabhängig vom Grad einer Hypoxämie aufzutreten [3]. Weitere Symptome beinhalteten Schmerz, Angst und Übelkeit, während Husten kaum oder gar nicht auftrat. Eine typische Opioiddosis in den letzten 24 h des Lebens waren 15 mg Morphin subkutan, kontinuierlich über einen Perfusor verabreicht [16]. Der Einsatz von Opioiden erscheint also effizient bereits in niedrigen Dosierungen, auch bei COVID-19, und im Sinne einer adäquaten Symptomkontrolle wichtig für die Patient:innen.

### Große Unterschiede *innerhalb* und *außerhalb* der Palliativmedizin in unserer Umfrage

Ärzt:innen und Pflegekräfte betrachteten den Umgang mit M/O, *innerhalb* der PM auch beim Thema Atemdepression, als sicherer im Vergleich zu *außerhalb* der PM. Dieser Trend zeigte sich ebenfalls bei Unruhe, Angst/Panik, und der Erleichterung des Sterbeprozesses. Ein besonders großer Unterschied bestand für die Dyspnoe: Nur die Hälfte der TN meinte, dass M/O zur Behandlung von Dyspnoe *außerhalb *der PM eingesetzt werden würde.

Diese Unsicherheit, insbesondere im außerpalliativmedizinischen Bereich, könnte assoziiert sein mit der Inkonsistenz der o. g. Leitlinien außerhalb der Palliativmedizin.

Ein deutlicher Warnhinweis war die Bewertung bei 17 % der TN, dass Morphin außerhalb der PM gezielt eingesetzt werde, um das Sterben zu beschleunigen (Abb. 5a). Allerdings kann dabei nicht differenziert werden zwischen einer Intention der Beschleunigung im Sinne von aktiver Sterbehilfe oder nur eine Inkaufnahme eines potenziell schnelleren Versterbens im Rahmen der Symptomkontrolle. Die Inkaufnahme nach dem Prinzip des „doppelten Effekts“ wäre in der ethischen Bewertung akzeptabel, wenn z. B. Morphin zur Symptomkontrolle eingesetzt wird, dabei aber auch das Risiko akzeptiert wird, dass dadurch zu einem früheren Versterben beigetragen werden könnte [9]. Eine beabsichtigte Beschleunigung wäre hingegen ethisch und juristisch nicht akzeptabel. Allerdings ist bei einer sachgerechten und individuell angepassten Titration mit Opioiden bei sterbenden Menschen mit Schmerzen nicht zu erwarten, dass sie vorzeitig an einer solchen Opioidgabe versterben.

Eine retrospektive Studie stationär eingewiesener Patienten, die eine reine Symptomkontrolle und Änderung der Opioiddosis erhielten, zeigte keinen signifikanten Unterschied bezüglich der Zeit bis zum Versterben in der Niedrigdosis- versus Hochdosisgruppe [1]. Das Einbeziehen einer palliativmedizinischen Konsultation hingegen erhöhte die Überlebenszeit sogar signifikant in dieser Studie. Dies weist darauf hin, dass ein schnelleres Versterben durch Opioidgabe unwahrscheinlich ist.

### Ideen zur Verbesserung der Versorgung

Prinzipiell sind Vorbehalte gegenüber dem Einsatz von Opioiden ein weltweit bekanntes Phänomen, das zu einer Unterversorgung von Patienten führen kann [7]. Dazu kommen Aspekte wie Assoziation von Morphin mit Abhängigkeit, Sterben [10] und dem Nicht-Aushalten von Leid [29]. Da in der SARS-CoV-2-Pandemie eine starke psychische Belastung des medizinischen Personals besteht, kann die Konstellation aus Vorbehalten, Unsicherheiten und auch heterogenen Leitlinien verschiedener Fachgesellschaften die medizinische Versorgung und angemessene Symptomkontrolle von Patienten mit COVID-19 zusätzlich erschweren. Die Unvorhersagbarkeit des Verlaufs einer COVID-19-Erkrankung stellt eine weitere Unsicherheit dar. Ein Teil der COVID-19-Erkrankten leidet unter Dyspnoe, während diese bei anderen fehlt [3, 17, 23]. Eine adäquate Einschätzung der betroffenen Patienten ist also per se eine klinische Herausforderung und muss aufgrund des dynamischen Verlaufs dieser Erkrankung immer wieder neu erfolgen.

Die beschriebenen Unterschiede können durchaus eine Versorgungslücke für Patient:innen mit Luftnot und insbesondere auch mit COVID-19 bedeuten. Einen Ansatz, diese Versorgungslücke zu schließen, stellt die Entwicklung evidenzbasierter, einheitlicher und fachübergreifender Leitlinien dar, um Widersprüchlichkeiten oder fachabhängige „blinde Flecken“ zu vermeiden. Im Bereich der Onkologie und PM gibt es bereits umfassende Empfehlungen für die Therapie der Dyspnoe (hier: „Atemnot“), die z. B. allgemeine mit nichtpharmakologischen Maßnahmen und Therapien der behandelbaren Ursachen kombinieren [22, 28]. Opioide haben hier trotz des Off-Label-Einsatzes einen relevanten Stellenwert.

Eine individuelle und wirksame Symptomkontrolle stellt eine der vielen Herausforderungen einer Pandemie dar. Palliativmediziner:innen fühlten sich in unserer Umfrage im Umgang mit M/O sicher, beurteilen diesen Umgang für nicht palliativmedizinische Fachbereiche jedoch als deutlich unsicherer: Dieses Ergebnis untermauert die Bedeutung des Einbeziehens eines palliativmedizinischen Konsildienstes, der auch von den meisten Umfrage-TN gewünscht wurde und in der Literatur empfohlen wird [23]. Im ambulanten Bereich könnten Teams der allgemeinen und spezialisierten Palliativversorgung als Ansprechpartner:innen zur Verfügung stehen. Fadul et al. [14] fordern hierzu explizit eine Integration von Palliativmediziner:innen in die COVID-19-Pandemie-Planung. Deren Einbindung in Entscheidungsalgorithmen und in Schulungsmaßnahmen zur Symptomkontrolle sollten dabei im Vordergrund stehen, ebenso die Begleitung des medizinischen Personals. Sie betrachten die Erhaltung der Arbeitsfähigkeit vor dem Hintergrund psychisch belastender Situationen und Entscheidungen als vorrangig.

Darüber hinaus müssen Aus, Fort- und Weiterbildungsmaßnahmen kurz-, aber auch langfristig die Vermittlung von Kenntnissen und Fertigkeiten im Umgang mit Opioiden noch klarer berücksichtigen [13, 25]. Kausale und symptomatische Therapieansätze – vor allem mit Opioiden – können sowohl bei kurativen als auch bei palliativen Behandlungsabsichten auch kombiniert gegen Luftnot zum Einsatz kommen. Dabei ist die subkutane Applikationsform eine einfache und wirksame Methode zur Gabe von Opioiden. Auch in unserer Umfrage fiel auf, dass die subkutane Gabe mit 89 % i*nnerhalb* der PM versus 36 % *außerhalb* der PM mehr als doppelt so häufig genannt wurde. Gerade bei Patienten in der letzten Lebensphase kann diese Applikation vorteilhaft sein.

### Zusätzliche Erkenntnisse zum Umgang mit M/O

Unruhe, Angst/Panik und Erleichterung des Sterbeprozesses sind keine anerkannten Indikationen für Opioide [6], auch wenn sich in der Literatur Hinweise für einen sinnhaften Einsatz über die klassischen Indikationen hinaus finden: z. B. bei Agitation, die ein Zeichen für Schmerzen sein kann [18]. Eine nicht-indikationsgemäße Anwendung schnell freisetzender Fentanyl-Formulierungen war vielen TN schmerzmedizinischer Fortbildungen in einer stichprobenartigen Fragebogenerfassung wie folgt bekannt: „im eigenen Patientengut“ 25 %, „bei mitbehandelnden Kollegen“ 25 %, „berichtsweise“ 40 % [31].

Auch wird in der Literatur diskutiert, ob Morphin bei einem Teil der COVID-19-Erkrankten Stress reduzieren kann [27]. Hier sollten im klinischen Setting in ergänzende und alternative Konzepte wie Benzodiazepine zur Behandlung von Dyspnoe und Unruhe erörtert werden. Studien zur Frage, inwieweit Opioide sich womöglich prognostisch auf das Immunsystem bei COVID-19-Erkrankten auswirken könnten, wären zukünftig wünschenswert. Ein im Jahr 2020 publiziertes systematisches Review zu Patienten mit chronischen Nicht-Krebsschmerzen stellte lediglich mit einer „sehr schwachen Evidenz“ von „niedriger Qualität“ fest, dass sich eine Langzeit-Opioidbehandlung auf das Immunsystem auswirken könnte [12]. Aufgrund fehlender Konsistenz in der Outcome-Erfassung war keine Metaanalyse möglich.

### Limitationen

Die Rücklaufquote war mit 8,2 % relativ niedrig, und die Ergebnisse sollten daher vorsichtig interpretiert werden. Niedrige Beteiligungen an Online-Umfragen werden häufig berichtet [4]. So war der häufigste Grund für eine Nichtteilnahme unter Allgemeinmedizinern deren Eindruck, von zu vielen Umfragen überschwemmt zu werden, und die Problematik begrenzter Zeitressourcen [24].

Anhand einer Drop-out-Analyse auf der Basis vorhandener Paramater in der DGP Datenbank – wie der Berufs- und Altersstruktur der DGP Mitglieder – konnten wir eine gute Vergleichbarkeit herleiten und halten daher eine Repräsentativität der Stichprobe für naheliegend. Für valide Ergebnisse spricht, dass die Erkenntnisse dieser Studie die Ergebnisse z. B. der qualitativen Studie von Charalambous et al. ergänzen, in denen die Unsicherheit im Umgang mit Opioiden deutlich wurde: Ärzt:innen und Patient:innen assoziierten Morphin mit Tod/Erkrankung im Endstadium und Abhängigkeit [10].

Schwierig einzuschätzen ist, inwieweit das Verständnis einiger Formulierungen unterschiedlich interpretiert worden ist: Der Bereich „*innerhalb* der PM“ könnte z. B. das eigene PM Team betreffen, aber auch andere PM-Bereiche, während „*außerhalb* der PM“ sich auf sämtliche andere Fachbereiche bezieht und sehr heterogen verstanden worden sein kann. Zusammengefasst sollte die Bereichszuordnung als grobe Einschätzung eingeordnet werden. Darüber hinaus sind die Aussagen für den Bereich außerhalb der PM nur indirekt über die Mitglieder der Palliativgesellschaft erhoben worden.

Morphin wurde im Fragebogen stellvertretend für Opioide genannt (s. a. „Studiendesign und Untersuchungsmethoden“). Diese Festlegung wurde lt. Rückmeldungen einzelner TN unterschiedlich wahrgenommen, da es an einigen Kliniken teils etablierte Konzepte mit anderen Opioiden gibt. Es könnte also sein, dass die konkrete Nennung nur dieses einen Opioids zu einer womöglich leicht veränderten Beantwortung führte.

Wir hatten Morphin gewählt, da es unserer Einschätzung nach das am häufigsten angewandte Opioid in der Klinik sowie in der hier referierten internationalen Literatur ist. So sollte für die Umfrage-TN ein konkreter Bezug zu konkreten Situationen im Klinik‑/Praxisalltag leichter hergestellt werden können.

In den Fragen zu Abb. 4a, b wurden unbeabsichtigt Mehrfachnennungen im Online-Fragebogen nicht ausgeschlossen, sodass zur Frage der Abb. 4a 9 bzw. 6 TN und bei Abb. 4b 15 bzw. 12 TN doppelte Angaben machten. Dies hat jedoch nur zu diskreten Änderungen der Häufigkeiten geführt.

## Fazit für die Praxis

- Die Mitglieder der DGP nahmen ein hohes Maß an Unsicherheit im Umgang mit Opioiden zur Symptomkontrolle *außerhalb* der PM wahr, insbesondere bezüglich des Umgangs mit M/O bei COVID-19-Erkrankten. Ihren eigenen Bereich schätzten sie bezüglich der Klarheit zum Einsatz von Opioiden als sicher ein und sahen ihn als gut geregelt an. Die Ergebnisse bestätigen die von den Teilnehmern vorgebrachte Notwendigkeit einer stärkeren Einbindung der spezialisierten Palliativeinrichtungen in die Planungen und Organisation der Behandlung von Patienten mit Covid-19.
- Die Entwicklung evidenzbasierter, einheitlicher und fachübergreifender Leitlinien ist erforderlich, um eine bestmögliche Versorgung COVID-19-Erkrankter unter Einbindung von palliativmedizinisch erfahrenen Behandelnden zu gewährleisten.

## Footnotes

1 Deutsche Gesellschaft für Palliativmedizin (2020) Persönliche Kommunikation: Mitgliederzahl und -struktur.
